# High Prevalence of Hepatitis C Virus Genotype 1b Infection in a Small Town of Argentina. Phylogenetic and Bayesian Coalescent Analysis

**DOI:** 10.1371/journal.pone.0008751

**Published:** 2010-01-18

**Authors:** Marcelo D. Golemba, Federico A. Di Lello, Fernando Bessone, Fabian Fay, Silvina Benetti, Leandro R. Jones, Rodolfo H. Campos

**Affiliations:** 1 Cátedra de Virología, Facultad de Farmacia y Bioquímica, Universidad de Buenos Aires, Buenos Aires, Argentina; 2 Hospital Provincial del Centenario, Servicio de Gastroenterología y Hepatología, Universidad Nacional de Rosario, Rosario, Argentina; 3 Laboratorio Cibic-Rosario, Rosario, Argentina; 4 Division of Molecular Biology, Estación de Fotobiología Playa Unión, CC 15, (9103) Rawson, Chubut, Argentina; Niels Bohr Institute and Biological Institutes, Denmark

## Abstract

Previous studies in Argentina have documented a general prevalence of Hepatitis C Virus (HCV) infection close to 2%. In addition, a high prevalence of HCV has been recently reported in different Argentinean small rural communities. In this work, we performed a study aimed at analyzing the origins and diversification patterns of an HCV outbreak in Wheelwright, a small rural town located in Santa Fe province (Argentina).

A total of 89 out of 1814 blood samples collected from people living in Wheelwright, were positive for HCV infection. The highest prevalence (4.9%) was observed in people older than 50 years, with the highest level for the group aged between 70–79 years (22%). The RFLP analyses showed that 91% of the positive samples belonged to the HCV-1b genotype. The E1/E2 and NS5B genes were sequenced, and their phylogenetic analysis showed that the HCV-1b sequences from Wheelwright were monophyletic. Bayesian coalescent-based methods were used to estimate substitution rates and time of the most recent common ancestor (tMRCA). The mean estimated substitution rates and the tMRCA for E1/E2 with and without HVR1 and NS5B were 7.41E-03 s/s/y and 61 years, 5.05E-03 s/s/y and 58 years and 3.24E-03 s/s/y and 53 years, respectively. In summary, the tMRCA values, the demographic model with constant population size, and the fact that the highest prevalence of infection was observed in elder people support the hypothesis that the HCV-1b introduction in Wheelwright initially occurred at least five decades ago and that the early epidemic was characterized by a fast rate of virus transmission. The epidemic seems to have been controlled later on down to the standard transmission rates observed elsewhere.

## Introduction

More than 170 million people are infected with *Hepatitis C Virus* (HCV) worldwide. Chronically infected patients who develop chronic hepatitis may progress to liver cirrhosis and have an increased risk of developing hepatocellular carcinoma (HCC) [1], [2].

The HCV genome is a positive sense, single-stranded RNA molecule of around 9.6-kb. It encodes a single polyprotein that is proteolytically processed by a combination of cellular and viral proteases into structural and nonstructural proteins [1], [3], [4].

Based mainly on phylogenetic analyses, six major lineages, namely genotypes 1 to 6, have been identified. These groups are further subdivided into several subtypes [5], [6]. HCV genotypes 1a, 1b and 3a are distributed worldwide as a result of HCV transmission through blood transfusion, use of inadequately sterilized medical equipment and intravenous drug use [7]. However, a non-negligible proportion of HCV infections have an “undefined” route of transmission.

Previous studies in Argentina have documented a general prevalence of HCV infection close to 2% (Consenso Argentino de Hepatitis C, 2007). In addition, a high prevalence of HCV has been recently reported in different Argentinean small rural communities [8]–[12]. In this work, we performed a study aimed at analyzing the origins and diversification patterns of an HCV outbreak in Wheelwright, a small rural town founded in 1900, of approximately 5800 inhabitants, located in Santa Fe province (Argentina).

## Results

### Epidemiological Analysis

The prevalence of HCV infections was studied during 2004 in a total of 1814 individuals, which represented approximately 31% of Wheelwright's population. Out of these 1814 volunteers (median age 40±18.6 years), 716 (39%) were male and 1098 (61%) were women. One hundred and seven individuals (5.9%) were reactive for antibodies to HCV by EIA, and 72 out of these were PCR positive for HCV RNA. The 35 EIA positive-PCR negative samples were positive in a second EIA, and only 17 out of 35 were LIA positive. Consequently, eighteen out of 107 (17%) should be considered false positives for EIA screening method. Altogether, these results indicate an overall HCV prevalence of 4.9% (89/1814) (Table 1).

**Table 1:** Epidemiological data.

CHARACTERISTIC	Total
N° of Samples	1814
Age, years	40±18.6
Gender, Male: Female	716 (39%) : 1098 (61%)
EIA	107(+) : 1707(−)
PCR (+)	72
PCR(−)/LIA(+)	17
Total Prevalence	26 Male: 63 Female (4.9%)
HCV Genotype 1a:1b:2a (RFLP)	5/72 (7%): 64/72 (89%): 3/72 (4%)

The highest prevalence of HCV infections was observed in the group of people older than 50 years, with the highest levels found in individuals between 60–69 years (17%) and between 70–79 years old (22%) (Table 2).

**Table 2:** Anti-HCV EIA prevalence by age in a total of 1814 volunteer sorted by age and gender.

Age (years)	Total	Positives	Prevalence (%)	Males	%	Females	%
<50	1198	9	1	2/488	0	7/710	1
50–59	299	22	7	7/113	6	15/186	8
60–69	199	34	17	9/65	14	25/134	19
70–79	102	22	22	7/41	17	15/61	25
>80	16	2	13	1/9	11	1/7	14
**Total**	1814	89	4.9	26/89	29	63	71

The restriction fragment length polymorphism (RFLP) analysis indicated that 64 out of the 72 PCR positive samples were genotype 1b (89%), 5 were genotype 1a (7%) and 3 were genotype 2a (4%) (Table 1).

Risk factors statistically associated (significance level of 0.05) with HCV infection were: surgery [Prevalence ratios (PR): 3.6 [2.1; 6.2]), transfusions (PR: 3.4 [2.1; 5.7]), dental treatment (PR: 2.8 [1.5; 5.1]), injections (PR: 2.7 [1.7; 4.2]) and out-of-hospital vaccines (PR: 2.2 [1.4; 3.5]). No association was found between genotypes and transmission risk factors (data not shown).

### Phylogenetic Analyses

The RFLP analyses indicated that genotype 1b was, by far, the most prevalent among the samples from Wheelwright. Of the 64 samples genotype 1b determined by RFLP, 55 were PCR amplified in the two regions (E1/E2 and NS5B). These 55 sequences were used to describe the origin and diversification of the virus during the infection process in Wheelwright. Two sequences were only amplified in one of the two regions and were not included in the analysis, while the remaining seven could not be amplified.

**Figure 1:**
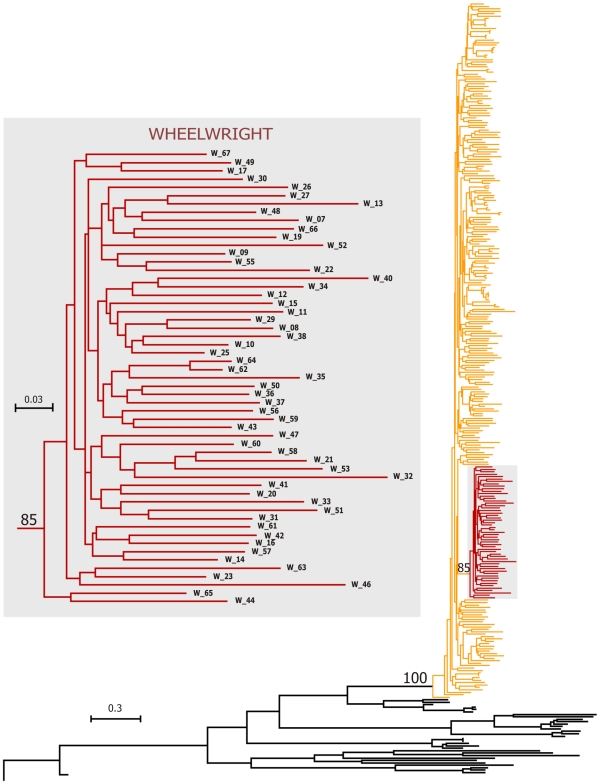
Maximum likelihood phylogeny of the concatenated analysis obtained with the PhyML software. A separation between sequences from the outbreak (red branches, n = 55) and those from other sequences can be observed in the shaded area to the right of the figure. Genotypes 1b (references sequences, n = 232) are represented by orange branches and the genotypes No-1b are represented by black branches (n = 34). A close up of the Wheelwright sequences are detailed on the left of the figure, with PhyML bootstrap support for the clade at 85%. Branch lengths reflect the likelihood distances.

The phylogenetic analyses were performed on both the separate, and concatenated, sequences from the E1/E2 and NS5B genes. In the separate analyses, the data from both E1/E2 and NS5B supported the monophyletic nature of the genotype 1b sequence from Wheelwright but with low bootstrap supports (data not shown). However, using concatenated sequences, all the methods used [maximum likelihood (ML), distance Neighbour-Joining, (NJ) and parsimony (P)] supported the monophyletic nature of the Wheelwright clade with good bootstrap values. The Wheelwright clade had a bootstrap value of 85 in the ML phylogenetic tree (Figure 1), and a value of 81 in the NJ one (supplemental Figure S1). The Parsimony analyses gave eight equally good trees (length = 18022). The bootstrap support for the Wheelwright clade in the Parsimony analyses was 63 (supplemental Figure S2).

### Molecular Evolutionary Rate and Divergence Times

The E1/E2 (both with and without HVR1) and NS5B partial sequence genes from the Wheelwright samples were used to estimate both the substitution rate and the tMRCA. Approximate marginal likelihoods of the different demographic models were calculated with enforced strict and relaxed molecular clocks (Table 3, 4 and 5).

**Table 3:** Estimates of the substitution rate and the tMRCA of HCV for the E2 gene with HVR1 by Bayesian coalescent methods, under several molecular clock and population genetic models.

Clock		Population Genetic Models				
		*Expansion*	*Exponential*	*Constant*	*Logistic*	*Skyline*
	Marginal likelihood^a^	−9804.12±0.39	*****	−9808.69±0.40	−9806.27±0.38	−9806.41±0.42
*Strict*	tMRCA (years)^b^	50 (17; 83)	*****	56 (23; 89)	55 (22; 87)	8 (3; 15)
	Substitution rate [10^−3^] c	4.17 (1.76; 8.18)	*****	4.16 (1.88; 7.49)	3.44 (1.65; 617)	23.31 (9.24; 43.02
	Marginal likelihood^a^	−9778.75±0.42	−9782.57±0.41	−9775.23±0.44	−9782.20±0.44	−9773.40±0.40
*Relaxed exponential*	tMRCA (years)^b^	52 (20; 84)	52 (18; 85)	61 (27; 94)	55 (25; 85)	24 (8; 43)
	Substitution rate [10^−3^] c	6.05 (2.55; 11.07)	5.30 (2.21; 9.87)	7.41 (3.01; 13.26)	4.26 (2.10; 7.07)	11.91 (4.83; 21.53)
	Marginal likelihood^a^	−9773.53±0.43	*****	−9777.07±0.41	−9776.99±048	−9778.11±0.46
*Relaxed lognormal*	tMRCA (years)^b^	47 (12; 79)	*****	56 (23; 88)	57 (27; 89)	8 (3; 13)
	Substitution rate [10^−3^] c	4.59 (1.66; 9.32)	*****	4.18 (1.83; 7.50)	3.32 (1.60; 5.60)	23; 86 (8.97; 44.15)

a Marginal likelihoods were calculated via importance sampling using the harmonic mean of the sampled likelihoods (with the posterior as the importance distribution).

b Time of the most recent common ancestor. The numbers between brackets are the 95% lower and upper values from the highest posterior density (95%HPD).

c Substitution rates are given in nucleotide substitutions per site per year (s/s/y). The numbers between brackets are the 95% lower and upper values from the highest posterior density (95%HPD).

***** = the model failed to converge.

**Table 4:** Estimates of the substitution rate and the tMRCA of HCV for the E2 gene without HVR1 by Bayesian coalescent methods under several molecular clock and population genetic models.

Clock		Population Genetic Models				
		*Expansion*	*Exponential*	*Constant*	*Logistic*	*Skyline*
	Marginal likelihood^a^	−6449.03±0.38	−6449.51±0.37	−6456.30±0.34	−6449.97±0.42	−6450.72±1.71
*Strict*	tMRCA (years)^b^	41 (10; 74)	43 (11; 75)	53 (21; 88)	52 (19; 81)	8 (3; 15)
	Substitution rate [10^−3^] c	3.28 (1.08; 7.22)	2.90 (1.00; 5.83)	3.33 (1.31; 6.18)	2.16 (0.98; 3.84)	14.71 (5.49; 27.20)
	Marginal likelihood^a^	−6446.23±0.45	−6450.92±0.43	−6443.15±0.38	−6451.68±0.41	−6444.57±0.85
*Relaxed exponential*	tMRCA (years)^b^	49 (17; 81)	46 (14; 79)	58 (26; 91)	51 (23; 82)	19 (4; 35)
	Substitution rate [10^−3^] c	3.77 (1.41; 6.97)	3.57 (1.23; 7.05)	5.05 (1.93; 9.56)	2.64 (1.17; 4.68)	8.27 (2.61; 14.59)
	Marginal likelihood^a^	−6442.08±0.42	*****	−6451.73±1.52	−6445.33±0.63	−6446.39±1.09
*Relaxed lognormal*	tMRCA (years)^b^	42 (9; 74)	*****	53 (23; 90)	45 (18; 77)	8 (3; 14)
	Substitution rate [10^−3^] c	3.31 (1.01; 7.51)	*****	2.82 (1.12; 5.20)	2.59 (1.11; 4.82	15.05 (5.89; 29.25)

a Marginal likelihoods were calculated via importance sampling using the harmonic mean of the sampled likelihoods (with the posterior as the importance distribution).

b Time of the most recent common ancestor. The numbers between brackets are the 95% lower and upper values from the highest posterior density (95%HPD).

c Substitution rates are given in nucleotide substitutions per site per year (s/s/y). The numbers between brackets are the 95% lower and upper values from the highest posterior density (95%HPD).

***** = the model failed to converge.

**Table 5:** Estimates of the substitution rate and the tMRCA for the NS5B gene by Bayesian coalescent methods under several molecular clock and population genetic models.

Clock		Population Genetic Models				
		*Expansion*	*Exponential*	*Constant*	*Logistic*	*Skyline*
	Marginal likelihood^a^	−2499.35±0.13	−2504.45±0.10	−2505.56±0.27	−2504.99±0.19	−2503.81±0.34
*Strict*	tMRCA (years)^b^	34 (4; 68)	34 (4; 63)	47 (15; 81)	42 (13.70)	42 (10; 75)
	Substitution rate [10^−3^] c	2.38 (0.53; 6.19)	2.02 (0.49; 5.01)	1.97 (0.68; 3.98)	1.41 (0.54; 2.75)	1.52 (0.46; 3.36)
	Marginal likelihood^a^	−2493.36±0.14	−2495.79±0.35	−2493.05±0.12	−2496.03±0.12	−2494.71±0.34
*Relaxed exponential*	tMRCA (years)^b^	40 (8; 73)	36 (8; 67)	53 (20; 87)	43 (10; 79)	12 (2; 25)
	Substitution rate [10^−3^] c	2.49 (0.65; 5.60)	2.08 (0.59; 4.60)	3.24 (1.09; 6.43)	1.73 (0.51; 3.76)	6.55 (1.71; 13.80)
	Marginal likelihood^a^	−2496.27±0.12	−2500.87±0.08	−2499.42±0.25	−2501.03±0.13	−2499.40±0.32
*Relaxed lognormal*	tMRCA (years)^b^	35 (5; 66)	35 (5; 68)	47 (14; 81)	40 (9; 75)	8 (2; 16)
	Substitution rate [10^−3^] c	2.22 (0.52; 5.60)	2.06 (0.47; 5.16)	2.07 (0.70; 4; 17)	1.63 (0.47; 3.80)	7.75 (2.33; 16.00)

a Marginal likelihoods were calculated via importance sampling using the harmonic mean of the sampled likelihoods (with the posterior as the importance distribution).

b Time of the most recent common ancestor. The numbers between brackets are the 95% lower and upper values from the highest posterior density (95%HPD).

c Substitution rates are given in nucleotide substitutions per site per year (s/s/y). The numbers between brackets are the 95% lower and upper values from the highest posterior density (95%HPD).

The Bayes Factor (BF) analysis favored the relaxed exponential molecular clock and the constant population size over the other models for both the E1/E2 and NS5B genes. However, these models were not significant with respect to the relaxed exponential molecular clock with Bayesian Skyline Plot (BSP), and the relaxed lognormal molecular clock with expansion growth model for E1/E2 with HVR1. Likewise, the relaxed lognormal molecular clock with expansion growth model was not significant for E1/E2 without HVR1. Based on these results, the relaxed exponential molecular clock and the constant population size (i.e., the models that use the fewest parameters) were selected to avoid over-parameterization.

In correspondence with the parametric model selected by the BF analysis, the BSP model (which estimates past population dynamics through time independently of any fixed parametric model of demographic history) indicated that the population size was maintained over time for the three data sets, under the different molecular clock models. However, the BSP model gives a more recent tMRCA and faster substitution rates than the other demographic models.

With the relaxed exponential molecular clock and the constant population size, the mean estimated substitution rates for E1/E2 with or without HVR1 and NS5B were 7.41E-03 (HPD95% = 3.01E-03; 13.26E-03), 5.05E-03 (HPD95% = 1.93E-03; 9.56E-03) and 3.24E-03 s/s/y (HPD95% = 1.09E-03; 6.43E-03) respectively (Table 3, 4 and 5). The density rate shows the marginal posterior density of the substitution rate for each of the runs under the different models (supplemental Figure S3).

Using these rates of nucleotide substitutions, the mean estimates for the tMRCA were 61 years (HPD95% = 27; 94) for the E1/E2 with HVR1, 58 years (HPD95% = 26; 91) for E1/E2 without HVR1 and 53 years (HPD95% = 20; 87) for NS5B (Table 3, 4 and 5, respectively). Supplemental Figure S4 shows the marginal posterior density of tMRCA for each of the runs under the different models.

## Discussion

Previous studies have documented a general prevalence of HCV of close to 2% in Argentina (Consenso Argentino de Hepatitis C, 2007). There are few previous reports of outbreaks of high prevalence of HCV in Argentina [8]–[11]. In the present work, a high prevalence of 4.9% (89/1814) was observed, mostly in the group of people older than 50 years, with the highest value for the group of people aged between 70 and 79 (22%). In a similar epidemiological study, Picchio et al. reported a prevalence of 5.7% for a small rural community of Argentina, geographically close to Wheelwright [8]. In our investigation, as in other studies [8]–[10], [13]–[16], the number of HCV-infected people coincides with the second pattern previously described by Wasley et al. where most infections are found among elder people [17].

A similar study performed in a small Sicilian town has been recently reported [14]. The authors also observed that intravenous drug addiction and sexual promiscuity are almost absent in this population, and the most probable HCV transmission route is the iatrogenic one. This type of transmission may also be the cause of the outbreak in Wheelwright.

The combination of phylogenetic analysis, molecular clock and demographic models forms a powerful framework to construct and test hypotheses about viral epidemics. In the phylogenetic analysis of the E1/E2 and NS5B regions extensively used for epidemiological studies [18]–[20], the trees obtained by different methods (ML, NJ and P) showed similar topologies, grouping the 55 HCV-1b-infected patients from Wheelwright in a single cluster. Taking the results of this study into account, it is reasonable to assume that all the genotype 1b Wheelwright sequences share a common ancestor, and that a single source of infection is responsible for the HCV epidemic spread.

Recently, a Bayesian relaxed-clock method that allows the implementation of sophisticated calibration methods has been published [21]. Using these methods, the divergence times and substitution rate were calculated for genotype 1b in Wheelwright. The exponential relaxed clock model and the constant population size performed better for the E1/E2 and NS5B genes with Wheelwright samples. Using this model, the estimated mean substitution rate from the NS5B region was 3.24E-03 s/s/y. This value is higher than the mean substitution rate values observed by others [22]–[26]. Part of this difference could be attributed to the different setups used in the different studies (e.g.: chimpanzees, a cohort of people infected with a common ancestor, intra-patients studies).

Higher substitution rates were determined using the E1/E2 region, both with, and without, the HVR1 (7.41E-03 s/s/y and 5.05E-03 s/s/y, respectively). These higher values may be attributed to the fast evolving characteristics of the genomic region that included three hypervariable regions.

The tMRCA based on the NS5B analyses was 53 years. Likewise, similar evolutionary time scales were obtained for the E1/E2 region without HVR1 (58 years), and the E1/E2 region with HVR1 (61 years). Although the tMRCA will not be the same as the date of introduction, especially in a population at constant size, our analysis allow us to speculate that the possible introduction and transmission events in Wheelwright started at least 50 years ago.

The molecular clock analyses give broadly similar results regardless of clock model and tree prior, with the exception of the BSP tree prior, which gives an intriguingly recent tMRCA, and faster substitution rates than the other analyses. The natural course of HCV infection comprises clinically silent periods in the most of the cases. Due to the inconspicuously nature of HCV infection, clinical manifestations of hepatic illness are often observed 20 to 30 years post-infection. However, in our case most of the hepatic illness were detected in patients older than 50 years and thus the results of the tMRCA from BSP model are at odds with epidemiological external data and could be confidently dismissed. It is possible that the underlying reason for the results could be attributed to the fact that the BSP model should be used only when the data are strongly informative about population history [27]. BSP places the least amount of constraint upon the data; in contrast, the parametric models possibly require less informative data given that they incorporate stronger priors on the analysis.

In summary, the HCV infection prevalence in Wheelwright is 4.9%. The phylogenetic analysis indicated a monophyletic origin for the HCV-1b epidemic. The tMRCA of the Wheelwright clade, the demographic model with constant population size, and the fact that the highest rate of infection was observed in elder people support the hypothesis that the HCV-1b introduction in Wheelwright initially occurred at least five decades ago, but were subsequently controlled, limiting further spread of the virus.

## Materials and Methods

### Studied Population

In 2004, the population of Wheelwright were encouraged to be tested for HCV infection. Blood samples were collected from 1814 volunteers, representing 31% of the population. These individuals were invited to undergo serological testing for HCV and complete a questionnaire aimed at identifying potential risk factors for HCV infection, such as surgery, injections, dental treatment, transfusions, out-of-hospital vaccines, accidental blood contact, job (present and past), intravenous drugs, tattooing, piercing, acupuncture, sexual abuse, jail and high-risk sexual behaviour such as multiple partners or men that have sex with men. Written informed consent to participate in this study was obtained from all patients. The ethics committee of the Universidad de Buenos Aires, Facultad de Farmacia y Bioquímica, approved this study protocol # Exp 701283.

### Serology: HCV Antibodies and RNA Detection

All samples were tested for Anti-HCV antibodies (Anti-HCV-Ab) by ELISA (EIA II-Cobas Core, Roche). The presence of HCV RNA was investigated by a home-made nested RT-PCR with a detection limit of 100 UI/ml in all positive Anti-HCV-Ab samples. A second blood sample was obtained from Anti-HCV-Ab positive/PCR negative patients, and both ELISA and PCR were repeated.

To further assess the presence of Anti-HCV-Ab in samples that were ELISA positive and PCR negative, an immunoblot confirmation assay was performed (INNO-LIA HCV AbIII Update, Innogenetics). Only EIA(+)/PCR(+) individuals and EIA(+)/LIA(+) were considered HCV positive in the seroprevalence study.

### HCV Genotyping

The HCV genotype was determined by RFLP analysis of the 5′UTR region as described elsewhere [28].

### Statistics

Prevalence ratios and 95% confidence intervals were calculated to estimate the degree of association of risk with HCV transmission.

### RNA Extraction, cDNA Synthesis and DNA Amplification for Phylogenetic Analysis

RNA was extracted from 100 µl serum using a commercial reagent (Trizol, Invitrogen). Reverse transcription (RT) reactions were carried out in 20 µl reactions. An aliquot of 9 µl of the eluted RNA and 20 ng random primers (Biodynamics) were incubated at 80°C for 5 min and 3 min at −20°C. Then, 2 µl of dNTP (10 mM), 100 units of M-MLV Reverse Transcriptase (Promega) and 20 units of Recombinant RNasin Ribonuclease Inhibitor (Promega) were added to the reactions and the mixtures were incubated at 37°C for 90 min and then kept at 80°C for 5 min to inactivate the enzyme.

The E1/E2 and NS5B genes were amplified by a hemi-nested PCR using Taq polymerase Recombinant (Invitrogen) with the following primers: E1/E2 external primers ES: (5′ GGA TAT GAT GAT GAA CTG GTC 3′, sense) and EA: (5′ RAA RCA RTC CGT GGG GCA 3′, antisense) and the internal primers: IS: (5′ TCC ATG GTG GGG AAC TGG GC 3′, sense) and EA: (5′ RAA RCA RTC CGT GGG GCA 3′, antisense) to generate a 672-nucleotide amplicon (containing the C-terminal of E1 and HVR1, HVR2 and HVR3) and NS5B external primers ES: (5′ GCC GTG ATG GGC TCC TCA TAC G 3′, sense) and EA: (5′ CCR GAT GCR TCG TGC GCG AC 3′, antisense) and the internal primers: ES: (5′ GCC GTG ATG GGC TCC TCA TAC G 3′, sense) and IA: (5′ GTA CCT AGT CAT AGC CTC CGT G 3′, antisense), which generate a 486-nucleotide fragment.

The amplified DNAs were purified from agarose gels using a commercial kit (QIAquik Gel Extraction Kit protocol, QIAGEN) and the amplicons were sequenced in both senses using the internal PCR primers.

### Phylogenetic Analysis

A partial region of the E1/E2 containing the C-terminal E1 and HVR1, HVR2 and HVR3 of the E2 (positions 1467 to 2024) and NS5B (positions 8202 to 8543) genes were sequenced from 55 samples from Wheelwright. These sequences were compared with the genotype 1b reference sequence (HCV-J, D90208) [29].

A strict requirement for dating an outbreak is to have a set of monophyletic strains (i.e., a group of sequences derived from a common ancestor). Current methods of phylogenetic analysis provide stringent tests of monophyly given that an adequate sample of sequences are used in the analyses. Essentially, the larger the portion of viral diversity represented in the dataset, the stronger the monophyly test for any group of sequences included in the dataset. Given that the outbreak in Wheelwright is largely dominated by 1b sequences, we downloaded 232 HCV-1b sequences from the Los Alamos sequence database for inclusion in the phylogenetic analyses. Tree rooting is also required to establish the monophyletic nature of a group of taxonomic units. Here, we used sequences from all the non-1b subtypes to root the tree by the “outgroup” criterion. The Wheelwright, reference 1b and outgroup sequences (n = 321) were aligned using the Clustal X program with default parameters [30].

Phylogenetic trees were constructed using distance (NJ), maximum likelihood (ML) and parsimony. The distance analyses were performed with the PAUP* v4.0b10 program [31]; the likelihood analyses with the PhyML v2.4.4 program [32] and the parsimony trees with TNT v1.1 program [33]. The ML and NJ trees were built using the GTR model, with a proportion of invariable sites and Γ-distributed rates across sites. Evolutionary models were inferred according to the Akaike Information Criterion (AIC) statistics [34] obtained with the Modeltest 3.7 program [35]. In the parsimony analysis we used multiple random addition sequences followed by tree bisection reconnection (RAS + TBR). Subsequently, several cycles of ratchet (R), tree-fusing (TF), tree-drifting (TD) and sectorial searches (SS) were performed until no improvements were observed in the tree lengths [36]. The robustness of the reconstructed phylogenies was evaluated by bootstrap analysis. The phylogenetic trees were analyzed using the Dendroscope v2.2 program [37].

### Molecular Evolutionary Rate and Divergence Times

Bayesian coalescent-based methods were used to estimate the substitution rate and the tMRCA, using sequences from the E1/E2 and NS5B genes. For the sequences of the E1/E2 gene, some modifications were necessary. The E2 gene contains 81 nucleotides corresponding to a hypervariable region (HVR1), which evolves faster than the surrounding sequences. Therefore, we performed independent analyses both with and without the inclusion of HVR1. The estimates of the rate of nucleotide substitutions per site per year (s/s/y) and the tMRCA (in years) were obtained by means of the Bayesian Markov Chain Monte Carlo (MCMC) techniques implemented in the BEAST v1.4.8 program [38]. Both strict and relaxed (uncorrelated lognormal and uncorrelated exponential) molecular clocks were enforced [38]. Five demographic models were applied as coalescent priors: constant population size, exponential growth, expansion growth, logistic growth and Bayesian skyline plot (BSP) [39].

The dates of isolation of the virus were considered as contemporaneous (isochronous). Since this is not sufficient to reliably estimate an evolutionary rate or the tMRCA, the tMRCA was inferred using a normal distribution with a mean value of 50 years and standard deviation (stdev) of 20 years as prior distribution, based on our prior belief about the history of the Wheelwright epidemic. To estimate the evolutionary rate we used a lognormal distribution as prior, with a lognormal mean of **−**6.603 s/s/y and a lognormal stdev of 0.711. The mean evolutionary rates were calculated from previously published data for the NS5B gene [22]–[24], [40]. In addition, since the expected rate for the E2 gene is considered in the prior distributions used for the NS5B, the evolutionary rate for the E2 gene was estimated using the same distribution lognormal values as for the NS5B gene.

These analyses were performed using the General Time Reversible substitution model [41] with gamma-distributed rates across sites and a proportion of sites assumed to be invariable (GTR+G+I). The length and number of MCMC chains were chosen so that the effective sample sizes (ESS) were above 100, indicating that the parameter space was sufficiently explored. The convergence of the parameters to a stationary distribution was assessed with the TRACER program [42], and the statistical uncertainties were summarized from the 95% highest probability density (HPD) intervals. Model comparisons were performed by a Bayes Factor analysis [43].

## Supporting Information

Figure S1 Neighbour-Joining phylogeny of the concatenated analysis obtained with the PAUP* software. A separation between sequences from the outbreak (red branches, n = 55) and those from other sequences can be observed in the shaded area to the right of the figure. Genotypes 1b (references sequences, n = 232) are represented by orange branches and the genotypes No-1b are represented by black branches (n = 34). Group of Wheelwright is detailed to the left of the figure with bootstrap supports equal to 81% from the same analysis. Branch lengths are proportional to the number of nucleotide substitutions.
(1.74 MB TIF)

Figure S2 Parsimony phylogeny of the concatenated analysis obtained with the TNT software. The strict consensus tree of parsimony analysis obtained from eight most parsimony trees. A separation between sequences from the outbreak (red branches, n = 55) and those from other sequences can be observed in the shaded area to the right of the figure in the strict consensus tree. Genotypes 1b (references sequences, n = 232) are represented by orange branches and the genotypes No-1b are represented by black branches (n = 34). Group of Wheelwright is detailed to the left of the figure with a bootstrap supports equal 63% from the same analysis.
(1.61 MB TIF)

Figure S3 The marginal posterior density of substitutions rate for independent MCMC runs under different models. A) E1/E2 gene with HVR1. B) E1/E2 gene without HVR1 and C) NS5B gene.
(3.60 MB TIF)

Figure S4 The marginal posterior density for the tMRCA for independent MCMC runs under different models. A) E1/E2 gene with HVR1. B) E1/E2 gene without HVR1 and C) NS5B gene.
(3.56 MB TIF)
