# Supplementary figures and images for: High Prevalence of Hepatitis C Virus Genotype 1b Infection in a Small Town of Argentina. Phylogenetic and Bayesian Coalescent Analysis
Source: PLoS One. 2010 Jan 18;5(1):e8751. doi: 10.1371/journal.pone.0008751 (PMC2807465; doi:10.1371/journal.pone.0008751)

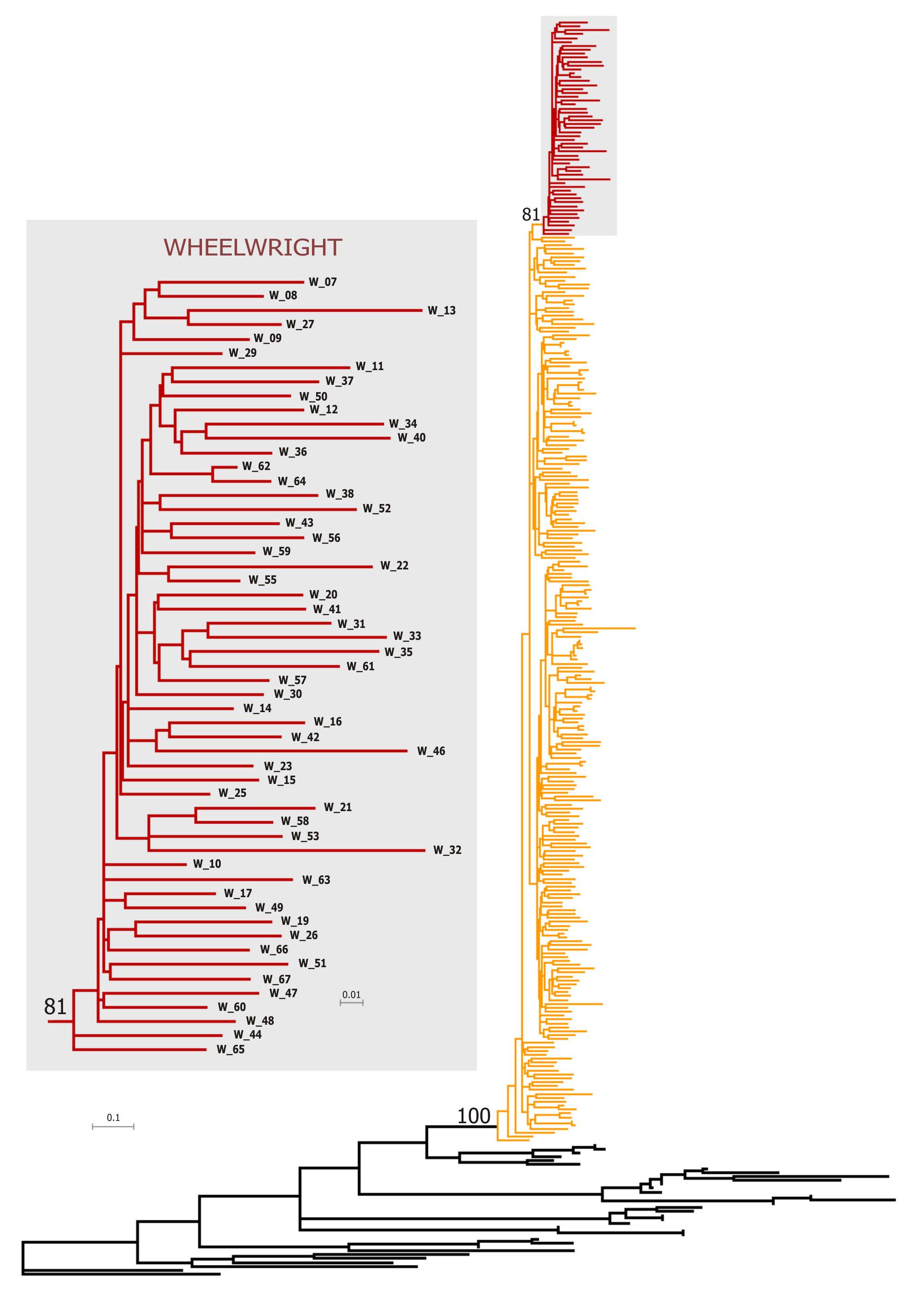

Supplement: Figure S1 — Neighbour-Joining phylogeny of the concatenated analysis obtained with the PAUP* software. A separation between sequences from the outbreak (red branches, n = 55) and those from other sequences can be observed in the shaded area to the right of the figure. Genotypes 1b (references sequences, n = 232) are represented by orange branches and the genotypes No-1b are represented by black branches (n = 34). Group of Wheelwright is detailed to the left of the figure with bootstrap supports equal to 81% from the same analysis. Branch lengths are proportional to the number of nucleotide substitutions. (1.74 MB TIF) [file pone.0008751.s001.tif]

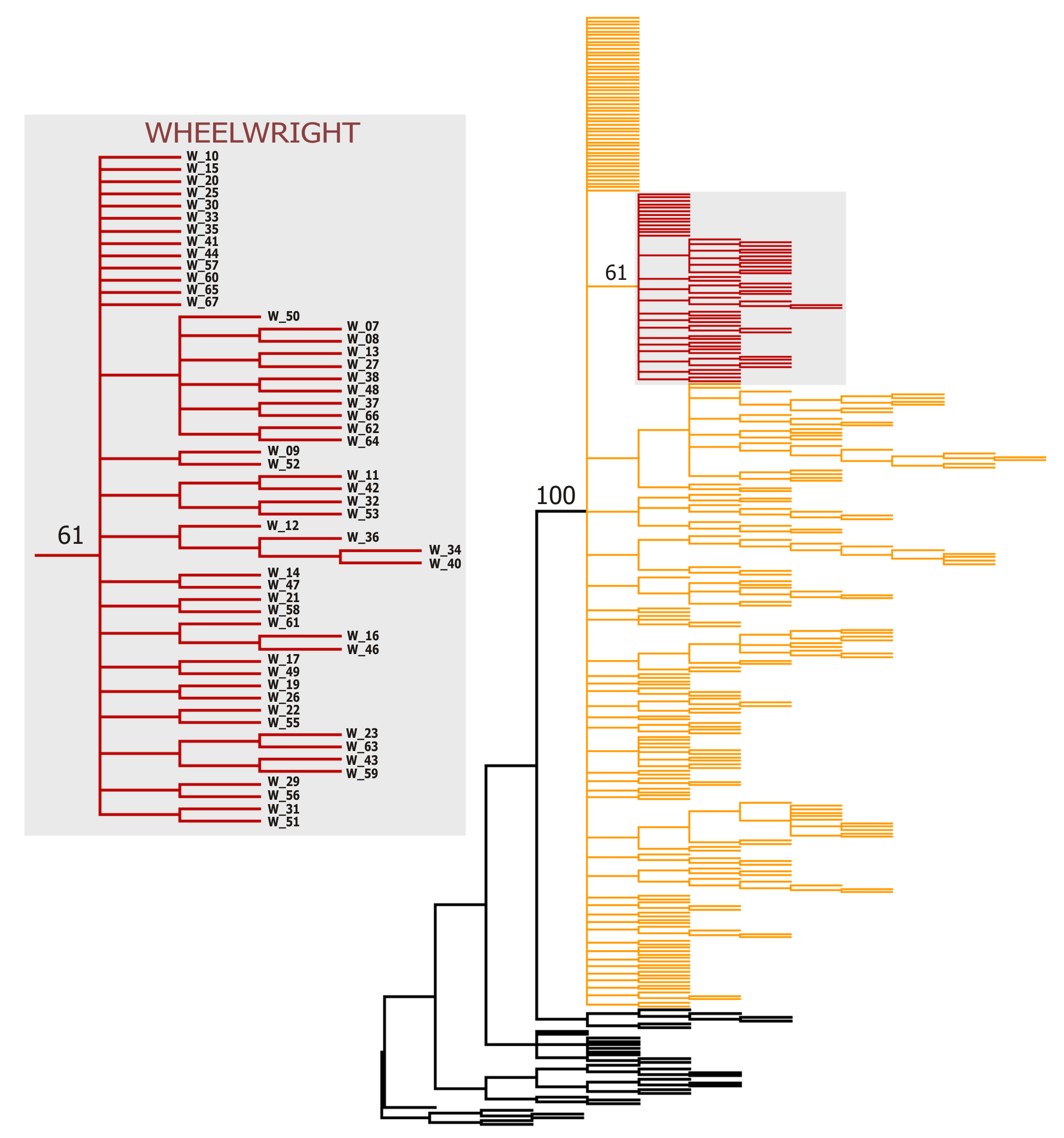

Supplement: Figure S2 — Parsimony phylogeny of the concatenated analysis obtained with the TNT software. The strict consensus tree of parsimony analysis obtained from eight most parsimony trees. A separation between sequences from the outbreak (red branches, n = 55) and those from other sequences can be observed in the shaded area to the right of the figure in the strict consensus tree. Genotypes 1b (references sequences, n = 232) are represented by orange branches and the genotypes No-1b are represented by black branches (n = 34). Group of Wheelwright is detailed to the left of the figure with a bootstrap supports equal 63% from the same analysis. (1.61 MB TIF) [file pone.0008751.s002.tif]

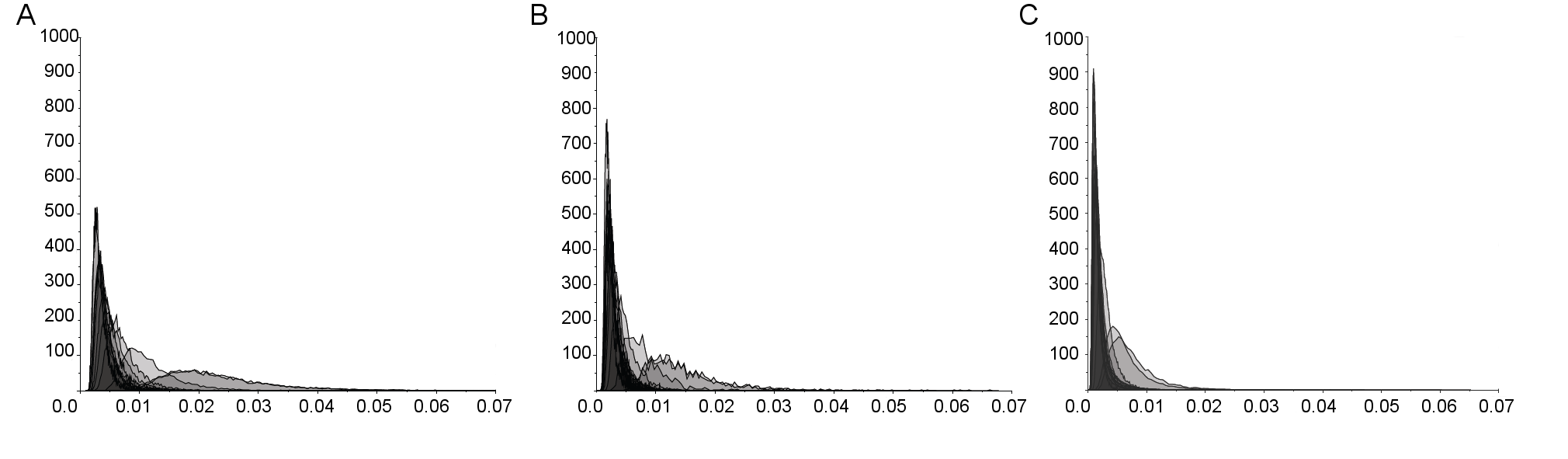

Supplement: Figure S3 — The marginal posterior density of substitutions rate for independent MCMC runs under different models. A) E1/E2 gene with HVR1. B) E1/E2 gene without HVR1 and C) NS5B gene. (3.60 MB TIF) [file pone.0008751.s003.tif]

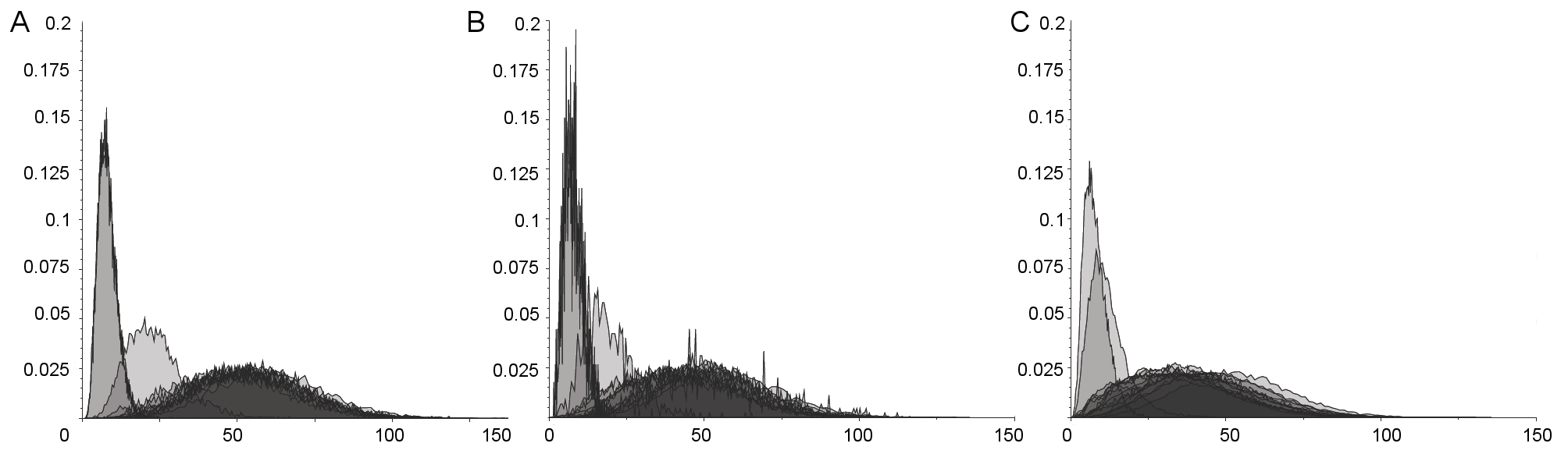

Supplement: Figure S4 — The marginal posterior density for the tMRCA for independent MCMC runs under different models. A) E1/E2 gene with HVR1. B) E1/E2 gene without HVR1 and C) NS5B gene. (3.56 MB TIF) [file pone.0008751.s004.tif]
